# Auditory Disturbances and SARS-CoV-2 Infection: Brain Inflammation or Cochlear Affection? Systematic Review and Discussion of Potential Pathogenesis

**DOI:** 10.3389/fneur.2021.707207

**Published:** 2021-08-04

**Authors:** Pietro De Luca, Alfonso Scarpa, Massimo Ralli, Domenico Tassone, Matteo Simone, Luca De Campora, Claudia Cassandro, Arianna Di Stadio

**Affiliations:** ^1^Department of Medicine, Surgery and Dentistry, University of Salerno, Salerno, Italy; ^2^Department of Sense Organs, Sapienza University of Rome, Rome, Italy; ^3^Otolaryngology Unit, San Giovanni Addolorata Hospital, Rome, Italy; ^4^Department of Surgical Sciences, University of Turin, Turin, Italy; ^5^Department of Surgery and Biomedical Sciences, Section of Otorhinolaryngology, “Santa Maria della Misericordia” University Hospital, Perugia, Italy

**Keywords:** COVID-19, hearing loss, SARS-CoV-2, brain inflammation, tinnitus, sudden hearing impairment

## Abstract

Patients affected by COVID-19 present a series of different symptoms; despite some of these are common, other less likely appear. Auditory symptoms seem to be less frequent, maybe because rarer or, alternatively, because they are underestimated during the clinical investigation. The hearing impairment might be related to the central or peripheral involvement of the auditory pathways; in particular, the likelihood of thrombosis might be one of the causes. To date, the prevalence of auditory symptoms such as sudden or progressive sensorineural hearing loss and tinnitus is unclear in COVID-19 patients. However, their presence might be an early sign of thrombosis or spread of the infection into the brain. In this systematic review of the literature we investigated the presence of auditory symptoms in COVID-19 patients and discussed their potential origin and causal relationship with SARS-CoV-2. Results showed that, despite rarely, auditory impairment can appear in patients with COVID-19 and should always be investigated for an early treatment and potential indicator of involvement of the central nervous system.

## Introduction

Coronavirus Disease 19 (COVID-19) has spread worldwide, negatively impacting the healthcare systems and institutions (1). COVID-19 presents several symptoms, that generally arise 2-14 days after the start of the infection. Common symptoms include fever, cough, shortness of breath, respiratory distress; furthermore, olfactory and gustatory alterations have been widely reported (2, 3). The Severe Acute Respiratory Syndrome Coronavirus 2 (SARS-CoV-2) infection is responsible of different neurological manifestations and systemic complication (4).

Despite rarely, several viral infections that determine inflammation of the cochlea can cause auditory deficits (5); the pathogenetic mechanisms of these symptoms have been widely explained and confirmed by the literature (6, 7). Researchers have attributed their onset to the peripheral damage affecting the cochlea (6) or to the involvement of the central auditory pathways (8).

Several studies have described the presence of brain lesions as responsible of auditory impairment (9–11), supporting the hypothesis that SARS-CoV-2, which has neuro-invasive characteristics, might determine a central hearing loss in COVID-19 patients both in the active phase and during recovery (12). It has been shown that the virus can spread from neuroepithelium to the olfactory bulb to the brain (13–15), causing loss of smell (16), persistent cough after pneumonia resolution (17), memory deficit (18), and neurocognitive problems (19).

Although the central hypothesis seems to be the most plausible, the peripheral involvement of the cochlea cannot be totally excluded.

The presence of auditory symptoms in COVID-19 might be underestimated because they are not a primary symptom of the disease, while they might be a sign of the spread of the virus in the superior auditory pathways, or of a thrombosis.

The aim of this paper is to assess the incidence of sudden sensorineural hearing loss (SSNHL) and hearing deterioration in COVID-19 patients and discuss their possible causes.

## Methods

**Figure 1:**
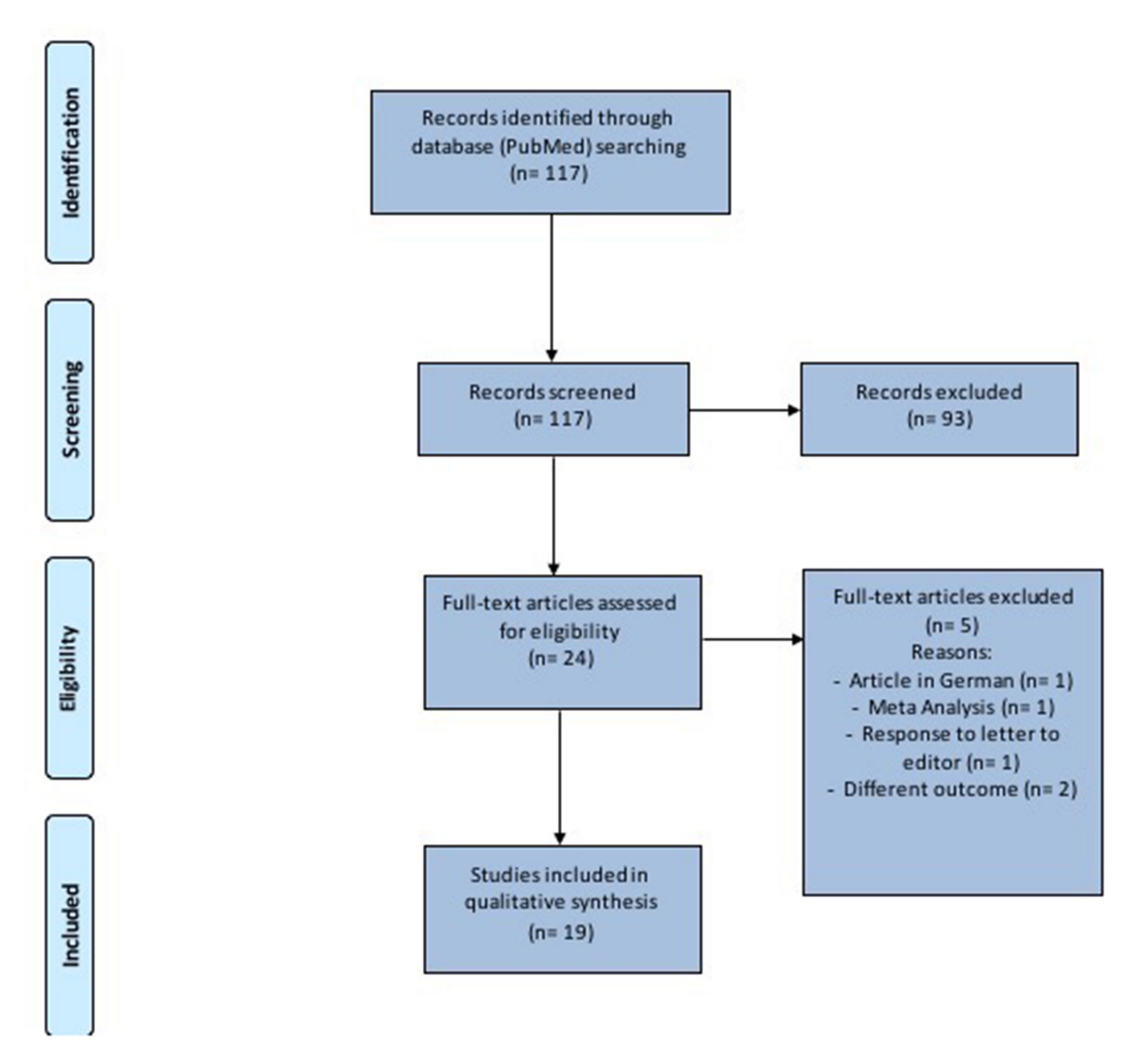
Prisma diagram to illustrate the method used to select the articles.

This study was performed in accordance with the Preferred Reporting Items for Systematic Reviews and Meta-analysis (PRISMA) checklist and statement recommendations (Figure 1). The nature of this review did not require Institutional Review Board approval.

## Search Strategy

A comprehensive search strategy, developed in partnership with a medical librarian, was performed on PubMed, Scopus and Google Scholar without time restrictions. The keywords used were: “hearing loss,” “hearing impairment” “tinnitus” “audio and vestibular symptom” “sudden hearing loss,” “SARS-CoV-2” and “COVID-19”. Only articles in the English language were considered for the analysis.

Two independent investigators reviewed the articles extracted from the literature review. Duplicates were removed, then each reviewer singularly filled in an Excel data sheet (Microsoft Corporation, USA) including information extracted from the articles. Files were then compared and disagreements on the inclusion/exclusion papers were debated until complete agreement of both researchers. Only papers that received full consensus were considered.

PRISMA guidelines were followed to conduct the systematic review and the full list of references was screened for potentially relevant articles.

## Study Selection Criteria

We included articles with the following characteristics: patients (0-99 years) affected by sudden sensorineural hearing loss or hearing deterioration after SARS-CoV-2 infection, written in English language, with full-text available. There were no restrictions in terms of diagnostic tools used to detect SARS-CoV-2. Suspected/unconfirmed COVID-19 patients were excluded. Selected articles were read in full to assess the study objectives and the level of evidence.

## Data Extraction

A spreadsheet was filled using the data extracted from the articles read in full by the researchers. The following information were included: name of the author, year of publication, type of study, country where the study was conducted, number of subjects analyzed, patients' characteristics, auditory results, treatment, outcome, presence or absence of the comparison group, characteristics of control groups.

## Risk of Bias Assessment

The National Institutes of Health's (NIH) quality assessment tools were used to assess the risk-of-bias checklists due to the different study designs (20). The rating of each study was categorized as: poor, fair or good (i.e., unbiased and fully described). The two authors independently gave a score to each article and any disagreement was resolved by direct comparison among the researchers.

## Results

### Study Selection

One-hundred seventeen records were identified (Figure 1 and Table 1). After removal of duplicates and abstract evaluation, 93 articles were excluded. Twenty-four full-text articles matched the inclusion/exclusion criteria. Five full-text articles were excluded because at high-risk of bias and the remaining 19 were included in the systematic review. The articles identified an association between SARS-CoV-2 and hearing impairment/sudden sensorineural hearing loss. All studies were published over a period of 2 years, between 2019 and 2021.

**Table 1:** Summary of studies included in the systematic review.

**References**	**Design of the study**	**Overall quality rating consensus**
Sriwijitalai and Wiwanitkitb (21)	Case study	Poor
Degen et al. (22)	Case study	Fair
Lang et al. (23)	Case study	Fair
Lamounier et al. (24)	Case study	Good
Koumpa et al. (25)	Case study	Fair
Guigou et al. (26)	Case study	Fair
Jacob et al. (27)	Case study	Poor
Abdel Rhman and Abdel Wahid (28)	Case study	Fair
Kilic et al. (7)	Case series	Good
Perret et al. (29)	Case study	Fair
Gunay et al. (30)	Case study	Fair
Chern et al. (31)	Case study	Fair
Gallus et al. (32)	Cross-sectional	Fair
Alves de Sousa et al. (33)	Cross-sectional	Good
Karimi-Galougahi et al. (34)	Case series	Fair
Celik et al. (35)	Cross-sectional	Good
Mustafa (36)	Cross-sectional	Poor
Munro et al. (37)	Cross-sectional	Fair
Chirakkal et al. (38)	Case study	Fair

### Study Characteristics

#### Sudden Sensorineural Hearing Loss in Patients With SARS-CoV-2 Infection

Twelve full-text articles were identified (7, 21–31) (Table 2); we identified 11 case reports and 1 case series. Two studies were conducted in Turkey, two in France, two in the United Kingdom, and one in each of the following countries: Thailand, Germany, Brazil, Australia, Egypt, and United States of America. Sixteen patients (10 men and 6 women; age range 18-84 years) evaluated in these studies tested positive for COVID-19. All patients suffered from SSNHL; tinnitus was reported by four patients. Two patients suffered from vertigo, two had nausea or vomiting, and one was affected by ear fullness. Four subjects had bilateral impairment. Five patients had right SSNHL and five left SSNHL; in two studies the side of the affection was not specified. The hearing function was always (100%) assessed by pure tone audiometry (PTA); tympanometry was performed in three studies, speech audiometry and otoacoustic emissions in one, respectively. Two studies did not report the audiological assessment.

**Table 2:** Characteristics of studies exploring sudden sensorineural hearing loss (SSNHL) in patients with COVID-19 and included in the systematic review.

**References, nation**	**Sample size**	**Age, gender**	**Symptoms**	**Laterality**	**Audiological evaluation**	**Treatment**	**Outcome**
**Sriwijitalai et al**. **(****21****), Thailand**	1	NA, F	SSNHL	NA	NA	NA	NA
**Degen et al**. **(****22****), Germany**	1	60, M	SSNHL, tinnitus	Bilateral	Pure tone audiometry	Cochlear implant	NA
**Lang et al**. **(****23****), Ireland**	1	30, F	SSNHL, tinnitus	Right	Pure tone audiometry	Oral prednisone	No improvement
**Lamounier et al**. **(****24****), Brazil**	1	67, F	SSNHL, tinnitus	Right	Pure tone audiometry	Oral and intratympanic corticosteroids	Isolated improvement of 250 kHz in the right ear, and of 4, 6, and 8 kHz in the left ear
**Koumpa et al**. **(****25****), Great Britain**	1	45, M	SSNHL	Left	Pure tone audiometry	Oral and intratympanic corticosteroids	Partial improvement
**Guigou et al**. **(****26****), France**	1	29, M	SSNHL	Bilateral	Pure tone audiometry	Oral corticosteroids	Complete recovery
**Jacob et al**. **(****27****), Australia**	1	61, F	SSNHL	NA	NA	No treatment	Complete recovery
**Rhman and Wahid** **(****28****), Egypt**	1	52, M	SSNHL, tinnitus	Left	Pure tone audiometry	Intratympanic corticosteroids	Hearing improvement
**Kilic et al**. **(****7****), Turkey**	5	40.8, M	SSNHL	3 left, 2 right	Pure tone audiometry, timpanometry	Oral steroid	Complete recovery (*hearing status obtained by phone*)
**Perret et al**. **(****29****), France**	1	84, M	SSNHL, rotational vertigo, vomiting	Right	Pure tone audiometry, otoacustic emissions	Oral steroid	Progressive clinical recovery
**Gunay et al**. **(****30****), Turkey**	1	23, F	SSNHL	Bilateral	Pure tone audiometry, timpanometry	Oral steroid	Hearing improvement
**Chern et al**. **(****31****), United States of America**	1	18, F	Bilateral SSNHL, intermittent bilateral aural fullness, and vertigo with associated nausea and vomiting	Bilateral	Pure tone audiometry, speech audiometry, timpanometry	Oral steroid and subsequent intratympanic corticosteroids	Fluctuating sensorineural hearing loss in the right ear, and a severe to profound mixed hearing loss in the left ear

*SSNHL, Sensorineural Hearing Loss; NA, Not Available*.

Nine patients were exclusively treated by oral steroids, one subject with intratympanic steroid only; three patients were treated by combining oral steroid and intratympanic corticosteroids. In one case the treatment was not described. One patient, who did not recover, needed cochlear implant and only one patient recovered spontaneously.

Three patients completely recovered the auditory function (only one spontaneously) and five had a partial improvement; two studies did not mention the hearing outcome.

#### Hearing Loss in Patients With SARS-CoV-2 Infection

Seven full-text articles were identified (27, 32–36, 38) (Table 3). They included four prospective studies, one retrospective study, one case series, and one case report. The studies were conducted in Italy, Portugal, Iran, United Kingdom, Turkey, Egypt, and Qatar. A total of one 188 patients were evaluated. Ninety-three patients were males, 95 were females; age ranged from 0 to 82 years. Only one study did not report details about age and gender of the patients. Hearing ability was assessed by PTA by four authors, speech audiometry in one study, otoacoustic emissions in three and tympanometry in two articles. Only one author reported hearing capacity as “self-reported hearing loss” without objective assessment.

**Table 3:** Characteristics of studies exploring hearing loss (HL) in patients with COVID-19 included in the systematic review.

**References, nation**	**Sample size**	**Age (mean), gender**	**Study design**	**Symtoms, laterality**	**Audiological evaluation**	**HL (frequency)**
**Gallus et al**. **(****32****), Italy**	48	4537 F/11 M	Retrospective study	4 patients reported subjective HL and 1 with tinnitus reported persistent symptoms	Pure tone audiometry, tympanometry and cochleo-stapedial reflexes testing	Thresholds at pure tone audiometry and vHIT gain were within normality range in all post-Covid-19 patients
**Alves de Sousa et al**. **(****33****), Portugal**	60	6313 F/47 M	Prospective study	NA	Pure tone audiometry	Patients with COVID-19 showed worse mean auditory thresholds starting from 1000 Hz through higher frequencies, when compared to controls
**Karimi-Galougahi et al**. **(****34****), Iran**	6	324 F/2 M	Case series	Vertigo in 1 patientTinnitus in 4 patientsHL of right ear in 4 patients, in left ear in 2 patients	Pure tone audiometry	Only 1 patient with sudden hearing loss was tested positive for SARS-CoV-2 infection
**Celik et al**. **(****35****), Turkey**	37	018 F/19 M	Cross-sectional study on infants whose mother was pregnant between March 2020 and December 2020 and were born after the diagnosis of COVID- 19 during pregnancy	NA	Otoacustic emission and contralateral suppression of otoacustic emission	TEOAE: statistically significant difference was observed among the two groups at 3 kHz and 4 kHz. Contralateral suppression of OAE: statistically significant difference was identified in all frequencies. Suppression was more effective in all frequencies in the normal group than patient group. This difference was more significant at high frequencies (2,3 and 4 kHz)
**Mustafa** **(****36****), Egypt**	20	NA, NA	Prospective study	Asymptomatic patients who were confirmed positive for COVID-19	Pure tone audiometry, otoacoustic emissions	The high frequency pure-tone thresholds and the TEOAE amplitudes were significantly worse in the test group
**Munro et al**. **(****37****),Great Britain**	16	642 F/14 M	Prospective study	Self-reported hearing loss after COVID-19 infection	Self-reported symptoms	None
**Chirakkal et al**. **(****38****), Qatar**	1	35, F	Case report	Reduced hearing sensitivity in left ear	Pure tone audiometry, speech audiometry, impedance audiometry, otoacustic emission	Mild low-frequency sloping toward normal hearing at high frequencies. Speech audiometry was normal. OAE was referred as “pass” in both ears for middle and high frequencies (i.e, 1 k, 2 k, and 4 k Hz) and absent for low frequencies in left ear indicative of outer hair cells damage.

*NA, Not available; TEOAE, Transient Evoked Otoacustic Emission; OAE, Otoacustic Emission; vHIT, video-Head Impulse Test*.

### Discussion

The results of our systematic review showed that, despite uncommon, the hearing function might be affected by SARS-CoV-2 infection. Because of wide differences among the studies and the lack of clinical trials, we were not able to perform a meta-analysis to clarify the real prevalence of this symptom.

Gallus et al. (32) performed a retrospective study investigating the audiological and vestibular characteristics of 48 non-hospitalized patients affected by COVID-19; after two consecutive negative RT-PCR on nasopharyngeal swabs, the doctors analyzed patients' auditory and vestibular functions. All subjects were investigated by pure-tone audiometry, tympanometry, and cochleo-stapedius reflex. Four (8.3%) of them reported self-perception of hearing loss, in presence of normal hearing threshold at the time of testing.

Alves de Sousa et al. (33) in a prospective study showed that patients with light forms of COVID-19 had worse auditory thresholds at 1,000, 2,000, 4,000, and 8,000 Hz compared to healthy subjects, and the severity of hearing loss worsened in patients with moderate-severe forms of the disease. The results observed by Karimi-Galougahi et al. (34) and the report from Chirakkal et al. (38) confirmed the association between hearing deterioration and SARS-CoV-2 infection in a sample of five patients.

Celik et al. tested the auditory function on newborns from mothers who suffered from COVID-19 during pregnancy; 37 infants were studied by transient evoked otoacoustic emission (TEOAEs), distortion product otoacoustic emission (DPOAE), and contralateral suppression of otoacoustic emission (CLS OAE). The results were compared to healthy controls. The authors found statistically significant differences between TEOAEs (3,000 and 4,000 Hz) of infants exposed to COVID-19 infection during pregnancy compared to the ones of non-exposed newborns. Analyzing the results of CLS OAE, the authors observed similar significant statistical differences in all auditory frequencies (more significant at high frequencies) (35). Mustafa et al. used TEOAE to evaluate the hearing function in a cohort of asymptomatic COVID-19 patients; their results were compared to those of non-infected subjects. The authors identified that TEOAE amplitude was significant worse in SARS-CoV-2 positive subjects compared to the control (36). Munro et al. investigated the hearing functions of 138 adult patients with confirmed SARS-CoV-2 infection using a questionnaire; 16 (13.2%) patients referred changes in their hearing status after COVID-19 diagnosis; unfortunately, no audiological evaluation was performed to objectively assess the hearing in this cohort (37).

Sriwijtalaia and Wiwanitkit were the first to describe a correlation between COVID-19 and hearing loss (21); unfortunately, detailed data about patients' characteristic were missing. Similarly, Degen et al. (22) described a 60-year-old man with profound SSNHL and COVID-19, but they did not report the outcome after treatment. Lang et al. (23) described a 30-year-old woman with severe unilateral SSNHL treated with oral steroids without significative improvement of the symptom. Furthermore, Lamounier et al. (24) and Koumpa et al. (25) showed that patients (one case for each author) could obtain partial recovery of their hearing function combining oral and intratympanic steroids. Guigou et al. described a 29-year-old man with bilateral SSNHL and positive to SARS-CoV-2, who obtained complete recovery of the hearing after treatment with oral corticosteroids (26). Notably, in this patient the SSNHL was the presenting symptom of COVID-19.

Chern et al. showed a case of bilateral intralabyrinthine hemorrhage in an adult woman affected by COVID-19; the patient suffered from bilateral SSNHL, aural fullness and vertigo. The magnetic resonance imaging (MRI) showed bilateral intralabyrinthine hemorrhage, which was identified as the cause of the hearing symptoms. The patient partially recovered hearing function after treatment with high-dose oral prednisone and left intratympanic dexamethasone injection (31).

Perret et al. described a case of acute labyrinthitis and SSNHL (29); the patient was treated with oral prednisone and showed progressive recovery. The oral steroid treatment was effective also for treating the hearing impairment in a patient on peritoneal dialysis program who showed SSNHL as presenting symptom of SARS-CoV-2 infection (29). An improvement of the hearing capacity was also reported by Abdel Rhman and Abdel Wahid (30); in this case the patient was treated with intratympanic steroids.

Kilic et al., speculating that hearing loss could be a presenting symptom of COVID-19, performed a real-time polymerase chain reaction (RT-PCR) in five consecutive male patients presenting with unilateral SSNHL. Only one of these subjects was positive for SARS-CoV-2, and SSNHL positively responded to COVID-19-specific treatment in the SARS-CoV-2 (7, 39).

Finally, Jacob et al. observed a complete and spontaneous recovery of hearing in a 61-year-old woman with SSNHL and SARS-CoV-2 infection (27).

### Etiopathology of Hearing Impairment in COVID-19

In patients affected by COVID-19, hearing loss and hearing disturbances might be related to a central and/or peripheral involvement of the auditory pathways. The cause can be indirectly (e.g., thrombosis) or directly (e.g., viral spread) related to SARS-CoV2.

**Figure 2:**
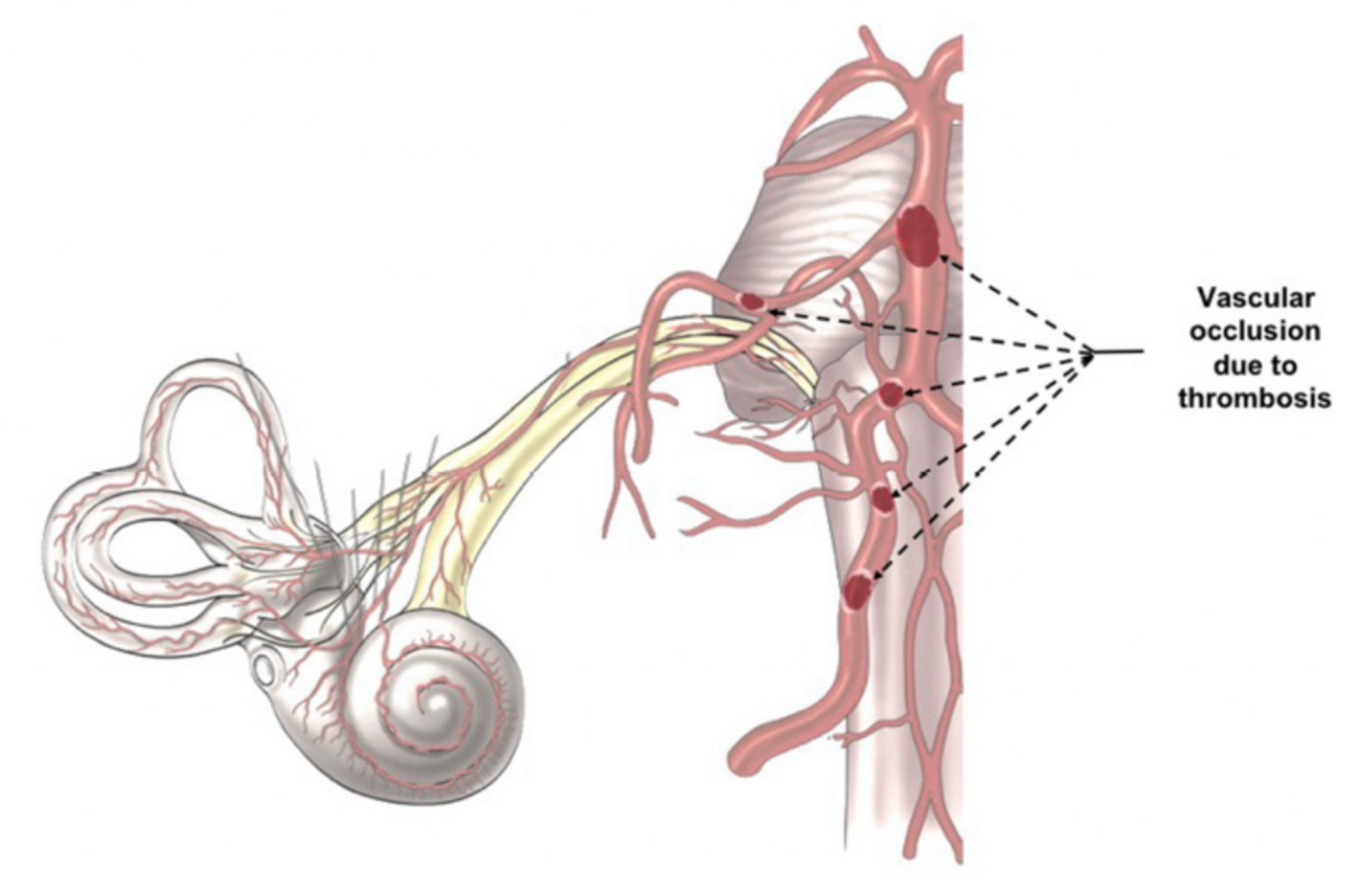
Indirect Virus Effect. The images illustrates the different position of a potential trombosis, which can determine the onset of the audio-vestibular disorders because it stops the blood flow in the audiovestibular artery.

SARS-CoV-2 infection increases the risk of systemic thrombosis (40), a condition that may determine neurological symptoms (41). Thrombosis is more common in the mild/moderate forms of the disease (42) rather than in severe ones, probably because in the latter anti-coagulant therapy is promptly administered (42, 43). An alteration of coagulation rate (44) could cause a macro and/or micro thrombosis, with consequent transitory ischemia and hypoxia in the auditory pathways determining the onset of hearing alterations. The thrombus, which can occlude the cochlear-vestibular artery or one of its afferent vessels (Figure 2), could determine transitory SSNHL or, in case of extremely rapid resolution, a slight hearing impairment or tinnitus. However, despite rapid resolution, transitory hypoxia into the cochlea could stress the inner ear cells and increase the concentration of reactive oxygen species (ROS), that could be responsible of additional damage of the hair cells. On the other hand, a thrombus in one of the vessels of the superior auditory pathways (Figure 2) can determine central hearing loss (45).

**Figure 3:**
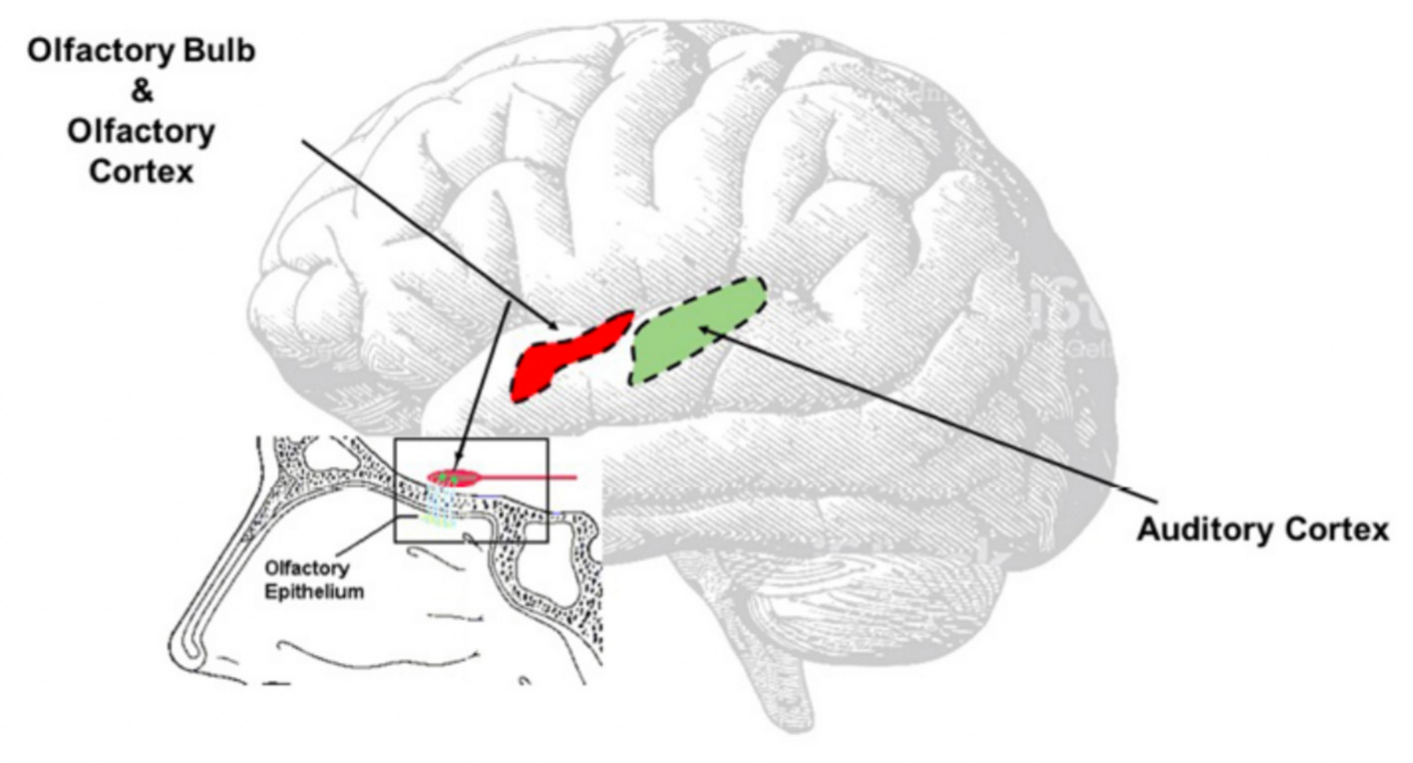
Direct Virus Effect. The image clearly shows the contiguity between the olfactory and the auditory areas. The virus can easy spread from the olfactory bulb to the olfactory area, reach the auditory area and once there inducing neuroinflammation responsible of the onset of the auditory symptoms.

Another hypothesis is that central auditory pathways—especially the auditory cortex (Figure 3)—might undergo the same inflammatory process observed in the olfactory area (16), directly caused by SARS-CoV-2. Recently, it has been confirmed that SARS-CoV2 spreads up to the olfactory bulb passing through the olfactory epithelium and lamina cribrosa (13); we speculate that the virus might, for contingency among the olfactory and auditory areas (Figure 3), determine transitory neuro-inflammation and consequent hearing symptoms. This hypothesis, although only speculative, could be supported by the clinical evidence of symptoms' resolution using steroids.

Moreover, the presence of vertigo/dizziness (46, 47) in patients with COVID-19 could have the same etiopathogenesis; in fact, a thrombosis in the audio-vestibular artery may alter the blood flow both in the cochlea and the vestibule explaining the presence of these symptoms (47). However, because of vestibular compensative mechanism, equilibrium disorders may be less perceived by the patients than hearing disturbances.

Vestibular disorders arising from central origin have been already confirmed in other diseases (48, 49); it is reasonable that SARS-CoV-2 spreading in the vestibular pathways may be responsible of equilibrium disorders observed in COVID-19 patients.

### Limits of the Study

This study presents several limitations. First, the sample size (204 patients) is small. Second, the quality rating of the studies is not always satisfactory; in fact, there are several case reports and the cross-sectional studies have uncontrolled designs or provide insufficient details on the control groups. Third, in some studies the hearing deterioration could be already present before SARS-CoV-2 infection. Lastly, some of these studies described “self-reported symptoms” without an objective assessment of the hearing.

### Conclusions

Hearing loss, despite rarely, might be present in COVID-19 patients. Auditory evaluation, although with all preventive measures to prevent contagion for healthcare providers, should be performed, especially if hearing disturbance are self-reported. The early recognition of these non-specific symptoms, which might be an early sign of brain inflammation, could help in preventing the spread of the infection to other areas of the brain.

## Data Availability Statement

The original contributions presented in the study are included in the article/supplementary material, further inquiries can be directed to the corresponding author/s.

## Author Contributions

AD: study concept and design, analysis of data, and article writing. PD and AS: systematic review and analysis of data. PD: article writing. MR: revision and criticism to the paper. CC, MS, LD, and DT: review of the literature. All authors approved the final version of the paper.

## Conflict of Interest

The authors declare that the research was conducted in the absence of any commercial or financial relationships that could be construed as a potential conflict of interest. The reviewer AF declared a past co-authorship with two of the authors AS and CC to the handling Editor.

## Publisher's Note

All claims expressed in this article are solely those of the authors and do not necessarily represent those of their affiliated organizations, or those of the publisher, the editors and the reviewers. Any product that may be evaluated in this article, or claim that may be made by its manufacturer, is not guaranteed or endorsed by the publisher.
