# Corrigendum: Auditory disturbances and SARS-CoV-2 infection: brain inflammation or cochlear affection? Systematic review and discussion of potential pathogenesis

**DOI:** 10.3389/fneur.2023.1234744

**Published:** 2023-06-26

**Authors:** Pietro De Luca, Alfonso Scarpa, Massimo Ralli, Domenico Tassone, Matteo Simone, Luca De Campora, Claudia Cassandro, Arianna Di Stadio

**Affiliations:** ^1^Department of Medicine, Surgery and Dentistry, University of Salerno, Salerno, Italy; ^2^Department of Sense Organs, Sapienza University of Rome, Rome, Italy; ^3^Otolaryngology Unit, San Giovanni Addolorata Hospital, Rome, Italy; ^4^Department of Surgical Sciences, University of Turin, Turin, Italy; ^5^Department of Surgery and Biomedical Sciences, Section of Otorhinolaryngology, “Santa Maria della Misericordia” University Hospital, Perugia, Italy

**Keywords:** COVID-19, hearing loss, SARS-CoV-2, brain inflammation, tinnitus, sudden hearing impairment

In the published article, there was an error in Figures 2, 3 as published. The figures were published in the incorrect order, so “Figure 2” should have been “Figure 3” and “Figure 3” should have been “Figure 2.” The corrected figures appear below.

**Figure 2:**
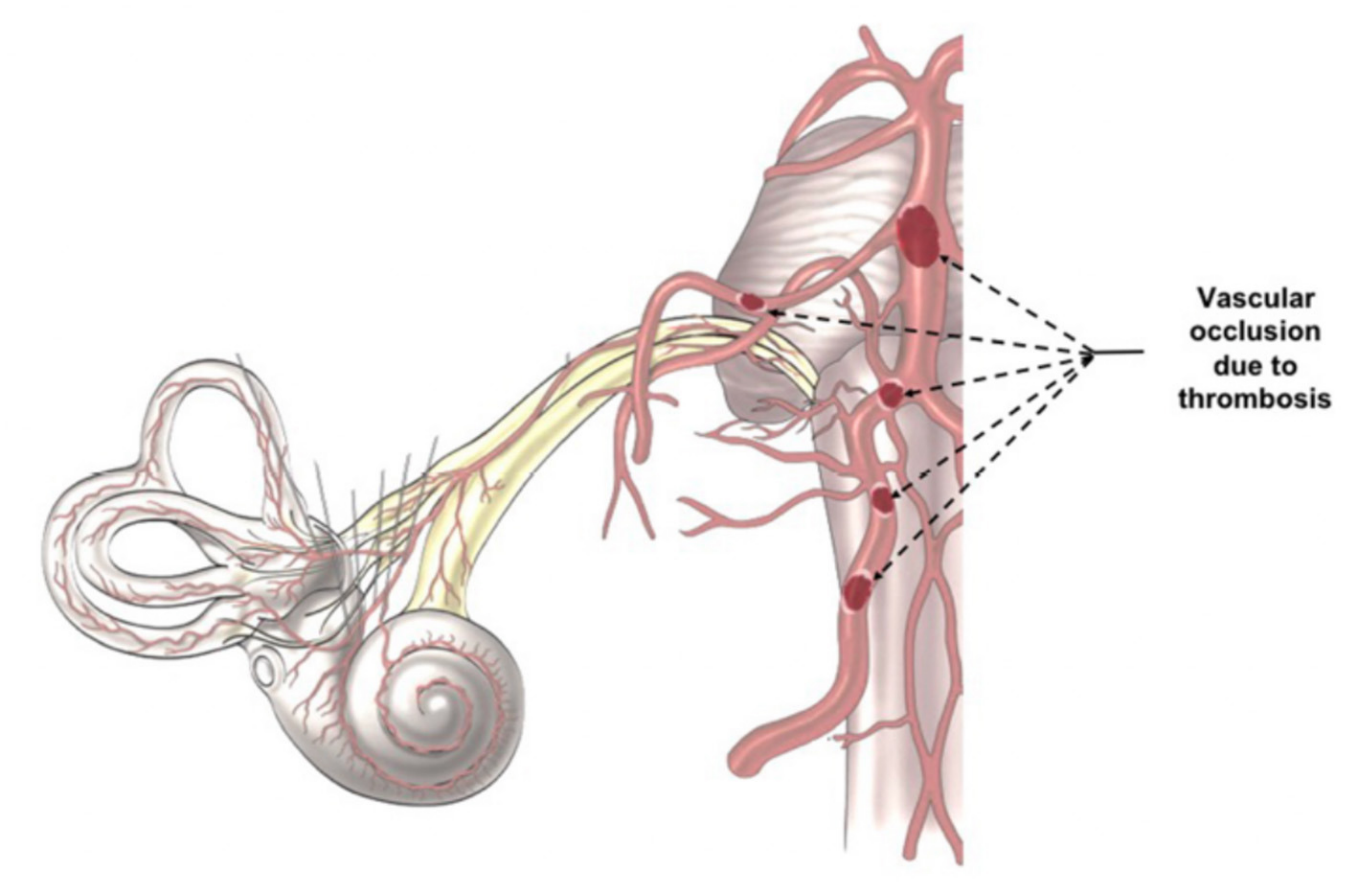
Indirect Virus Effect. The images illustrates the different position of a potential trombosis, which can determine the onset of the audio-vestibular disorders because it stops the blood flow in the audiovestibular artery.

**Figure 3:**
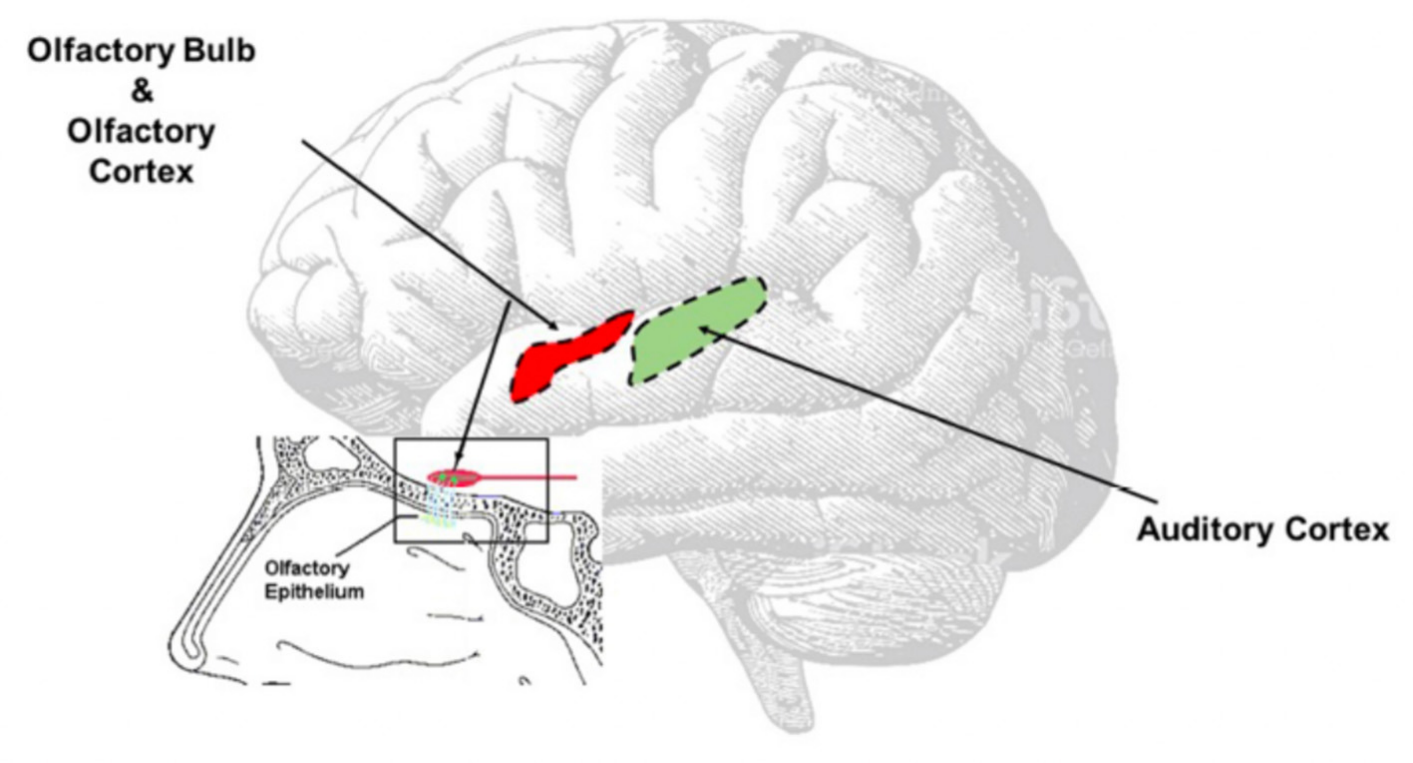
Direct Virus Effect. The image clearly shows the contiguity between the olfactory and the auditory areas. The virus can easy spread from the olfactory bulb to the olfactory area, reach the auditory area and once there inducing neuroinflammation responsible of the onset of the auditory symptoms.

The authors apologize for this error and state that this does not change the scientific conclusions of the article in any way. The original article has been updated.

